# snRNP: Rich Nuclear Bodies in *Hyacinthus orientalis* L. Microspores and Developing Pollen Cells

**DOI:** 10.1155/2009/209303

**Published:** 2009-06-25

**Authors:** K. Zienkiewicz, E. Bednarska

**Affiliations:** Department of Cell Biology, Institute of General and Molecular Biology, Nicolaus Copernicus University, 87-100 Toruń, Poland

## Abstract

The aim of the present work was the characterization of nuclear bodies in the microspore and developing pollen cells of *Hyacinthus orientalis* L.. The combination of Ag-NOR, immunofluorescence and immunogold techniques was used in this study. The obtained results showed the presence of highly agyrophylic extranucleolar bodies in microspore and developing pollen cells, which were finally identified as Cajal bodies. In all cases, a strong accumulation of snRNP-indicating molecules including TMG cap, Sm proteins and U2 snRNA, was observed in the examined nuclear bodies. In contrast to their number the size of the identified structures did not change significantly during pollen development. In the microspore and the vegetative cell of pollen grains CBs were more numerous than in the generative cell. At later stages of pollen development, a drastic decrease in CB number was observed and, just before anthesis, a complete lack of these structures was indicated in both pollen nuclei. On the basis of these results, as well as our previous studies, we postulate a strong relationship between Cajal body numbers and the levels of RNA synthesis and splicing machinery elements in microspore and developing pollen cells.

## 1. Introduction

Intron removing is one of the key steps in pre-mRNA processing. The splicing reaction occurs in large, ribonucleoprotein complexes, called spliceosomes. The basic elements of most spliceosomes are five classes of snRNPs, U1, U2, U4/U6 and U5, splicing factors from the SR family and about 300 different proteins [1].

Wide-scale use of immunocytochemical and in situ hybridization techniques in the last two decades enabled the localization of molecules involved in defined steps of gene expression in the cell. These methods allow us to study the distribution of different RNA types as well as the proteins participating in pre-mRNA splicing. The obtained results indicated that molecules involved in RNA transcription and processing are nonrandomly distributed in the cell nucleus. Based on these results, Spector [2] proposed what they called the “nuclear domains” idea. The term “nuclear domains” is used for describing the spatial and functional organization of gene expression in the nucleus. So far, three nuclear domains have been indicated as being involved in pre-mRNA splicing: (1) perichromatin fibrils (PFs), (2) interchromatin granules (IGs), and nuclear bodies termed as Cajal bodies or coiled bodies (CBs) [2–7].

Cajal bodies (coiled bodies) are the best characterized nuclear structures present in plant and animal cells. These spherical structures, with a diameter from 0.5 to 10 *μ*m, are present at a rate of 1 to 5 per nucleus of different cell types [8]. CBs exhibit high affinity to silver ions and their molecular marker is a phosphoprotein p80-coilin. The precise function of Cajal bodies remains unknown; however, with regard for their molecular equipment and frequent association with the genes encoding snRNA, snoRNA and histones, they are thought to have an important role in the expression of the mentioned genes. So far, it has been shown that Cajal bodies contain (1) molecules involved in rRNA metabolism (fibrillarin and nucleolin, U3, U8 and U14 snoRNAs), (2) transcription and pre-mRNA splicing factors (RNA pol II, TBP proteins, basal transcriptional factors TFIIF, TFIIH, all of the splicing snRNPs and Sm proteins), (3) molecules involved in transcription and processing of histone genes (U7 snRNA and SLBP protein), (4) cell cycle proteins (E/cdk cyclin), and (5) many other molecules such as topoisomerase I, lamin A, and RNA telomerase [8–11].

Cajal bodies are not the place of pre-mRNA splicing, since they are devoid of the SC35 splicing factor, pre-mRNA and U2AF^65^ [8]. However, because of the fact that CBs are especially rich in splicing machinery elements, including snRNPs and Sm proteins, it is suggested that they are an important element of the cellular splicing system. According to the hypothesis of Matera [12], transcription of snRNA encoding genes occurs at the periphery of CBs and newly formed snRNAs are targeted to the cytoplasm, where the Sm core domain is assembled and TMG cap is formed [3, 13]. Finally, snRNPs return into the nucleus and localize in Cajal bodies, where the methylation and pseudourydilation of these molecules take place [14]. Recently, an increasing number of reports have strongly suggested that Cajal bodies can also be the sites of miRNA and siRNAs biogenesis [15]. Thus, it seems that CBs are multifunctional nuclear structures that are involved in biogenesis, recycling and/or storage of molecules participating in key steps of gene expression in metabolically active eukaryotic cells. CBs are dynamic structures able to move from nucleolus periphery to nucleoplasm; thus they can be found as adjacent to or engrossed in the nucleolus as well as free in the nucleoplasm [9, 11, 16]. Moreover, their number and size depend on cell metabolic activity and the cell cycle [9, 16, 17].

Plant Cajal bodies are generally well characterized as being similar to animal CBs. They were shown to contain also molecules involved in pre-mRNA processing, including snRNPs and Sm proteins, as well as rRNA processing machinery, including U3 snoRNA and fibrillarin [17–20]. Thus, similar to animal cells, plant Cajal bodies seem to be related with nucleolar metabolism and impregnated by silver, in the same way as the fibrillar component of the nucleolus [20]. However, no DNA or rRNA was indicated in these structures in plant cells [21]. Visualization of CBs in living plant cells and *A. thaliana * plants transfected with a GFP-U2B” encoding fusion constructs showed that plant CBs are also dynamic structures within the nucleus with the ability to move, split and rejoin with each other [22]. Moreover, it was shown that the number of CBs in plant cells changes during the cell cycle and differentiation [8]. Given these facts, even in spite of the low similarity between vertebrate p80-coilin and recently identified plant Atcoilin [23], it generally seems that Cajal bodies are highly conserved nuclear structures and perform similar functions in all eukaryotic cells.

So far, there have only been a few reports relating to Cajal bodies in male gametophyte cells of plants. Identification of this type of nuclear bodies was reported in microsporocytes of larch [24], microspores of douglas [25] and larch as an element of bizonal bodies [26] as well as in microspores and pollen cells of *Brassica napus * [27, 28] and *Capsicum annuum * [28]. However, the latter studies were mostly focused on changes in Cajal body size and number accompanied by a microspore reprogramming to embryogenesis.

In the present study, we report the identification and molecular analysis of Cajal bodies during *Hyacinthus orientalis * L. pollen development. In our previous studies, we performed a detailed analysis of metabolic activity, including RNA synthesis and processing, in microspore and differentiating pollen cells of hyacinth [29–32]. Thus, we chose these cells as experimental models to check if and how the number of CBs is correlated with the levels of nuclear activity of microspore and developing pollen cells. Moreover, microspore and differentiating pollen grains are unique and excellent models for studies on the organization of gene expression, because they exhibit changing rates of RNA synthesis and undergo a physiological (natural) inhibition of transcription (mature pollen grain) during their development, in contrast to many other experimental systems, in which the metabolic activity of cells was artificially modulated using different kinds of inhibitors.

## 2. Material and Methods

### 2.1. Plant Material

Developing *Hyacinthus orientalis* L. microspores and pollen grains were used in the investigations. Bulbs of *Hyacinthus orientalis* L. cv Pink Pearl (Torseed, Torun, Poland) were planted in plastic pots and held in the refrigerator for 8 weeks at 6°C in the dark. Next, pots were removed to a growth-chamber, and each bulb was covered with a paper cone. Plants were grown at 26°C in 16-hour-light/8-hour-darkness (LD conditions) cycles (130 *μ*mol m^−2^s^−1^ cool white fluorescent tubes, *Polam *, Warsaw, Poland) for 2–4 weeks. Anthers, with differentiating pollen grains, were collected every 24 hours for 14 days. This time included all stages of a microspore and pollen grain development, previously described by Bednarska [29].

### 2.2. Ag-NOR Technique

For the Ag-NOR technique, anthers were fixed in Carnoy's solution. Subsequently a modified reaction according to Howell and Black [33] was performed. Fixed material was hydrated in decreased concentrations of ethanol, washed in 5% acetic acid and finally in distilled water. After maceration, anthers were placed on microscope slide covered with gelatin and cooled on dry ice for 15 minutes. Next, the material was incubated in a solution containing 2.5% gelatin (Difico Laboratories, Detroit, USA) and 50% water solution of AgNO_3_ with ratio 1 : 2 at room temperature for 15 minutes. The time of incubation was estimated basing on microscopic observations. After incubation, slides were washed with distilled water for 30 minutes and then treated with 5% sodium thiosulfate for 10 minutes. After washing with distilled water, material was dehydrated in increasing series of ethanol and embedded in Euparal. Samples were analyzed in a Nikon Eclipse 80i light microscope.

### 2.3. Quantitative Measurements

To calculate the number of nuclear bodies stained by AgNOR, 30 microspores and pollen grains in each stage from three different preparations were taken. Analysis was made using the Lucia General software (Laboratory Imaging, Prague, Czech Republic) compatible with a Nikon Eclipse 80i microscope equipped with a CPI Plan Fluor 100 (numerical aperture, 1.3) immersion oil objective.

To test differences among multiple samples (groups, i.e., number of nuclear bodies in different stages), a Kruskal – Wallis ANOVA test was used. The Mann-Whitney U test as the post hoc comparisons with Bonfferoni adjustments was computed to know what samples are particularly different from each other. All tests were performed with a *P*-value of less than .05.

### 2.4. Immunofluorescence Techniques

For immunofluorescence methods, anthers were fixed in the mixture of 4% paraformaldehyde and 0.25% glutaraldehyde in PBS overnight at 4°C. The material was dehydrated in increasing ethanol concentrations, supersaturated, and then embedded in BMM resin (butyl methacrylate, methyl methacrylate, 0.5% benzoin ethyl ether, 10 mM dithiothreitol; Fluka Chemie GmbH, Buchs, Switzerland). The embedded material was cut into semithin (1.5 *μ*m) sections, which were placed on microscope slides covered with Biobond (British Biocell International, Cardiff, UK). TMG snRNA was detected by incubating with a primary mouse anti-TMG antibody (Calbiochem, Bad Soden, Federal Republic of Germany) diluted 1 : 50 in 1% bovine serum albumin (BSA) prepared in PBS, pH 7.2, overnight at 4°C. Then samples were incubated with the secondary goat anti-mouse Alexa Fluor 488 (Molecular Probes) antibody diluted 1 : 500 in 1% BSA in PBS for 1 hour at 37°C. Sm proteins were detected by incubating with a primary mouse Y12 antibody diluted 1 : 50 in 1% bovine serum albumin (BSA) prepared in PBS, pH 7.2, overnight at 4°C. Next, samples were incubated with a secondary goat anti-mouse antibody conjugated with Cy3 fluorochrome (Sigma-Aldricht). DNA was stained with 4.6 diamidino-2-phenylindole (DAPI) (Fluka). Control reactions were performed by omitting of the primary antibodies (data not shown).

### 2.5. Fluorescence In Situ Hybridization

U2 snRNA was detected using the oligonucleotide probe TOR50, which is complementary to the first 20 nucleotides of U2 snRNA (Department of Bioorganic Chemistry, Centre of Molecular and Macromolecular Studies, Polish Academy of Sciences). The probe was chemically labelled with Cy3 fluorochrome at the 5′ end. After 1-hour prehybridization, FISH was performed for at least 12 hours at 37°C in a hybridization buffer (30% formamide, 4xSSC, 100 *μ*g/ml of herring sperm DNA). Then slices were washed in the 4xSSC, 2xSSC and 1xSSC, consecutively. DNA was stained with DAPI (Fluka). The control reaction was performed using the hybridization buffer with a sense probe.

Observations of slides were performed with a Nikon Eclipse 80i fluorescence microscope. The CPI Plan Fluor 100x (numerical aperture, 1.4) DIC H/N2 oil immersion lens and narrowband filters (UV-2EC, B- 2EC, G-2EC) were used. The results were registered with a Nikon DS-5Mc color cooled digital camera and the image-analysis Lucia G software (Laboratory Imaging, Prague, Czech Republic).

### 2.6. Immunogold Labeling

For immunogold techniques, the material was fixed as described above, dehydrated in alcohol and embedded in LR Gold (Sigma-Aldricht). The material was cut into ultrathin sections using *Leica * Ultramicrotome and placed on formvar-coated nickel grids. TMG snRNA was detected by incubating with a primary mouse anti-TMG antibody (Calbiochem, Bad Soden, Federal Republic of Germany) diluted 1 : 50 in 1% bovine serum albumin (BSA) prepared in PBS, pH 7.2, overnight at 4°C. Grids were then incubated with the secondary goat antimouse IgG antibodies conjugated with 10 nm gold (British BioCell) diluted 1 : 50 in 1% BSA prepared in PBS for 1 hour at 37°C. Sm proteins were detected by incubating with a primary mouse Y12 antibody diluted 1 : 50 in 1% bovine serum albumin (BSA) prepared in PBS, pH 7.2, overnight at 4°C. Then grids were incubated with the secondary goat antimouse antibody conjugated with 10 nm gold (British BioCell) diluted 1 : 50 in 1% BSA prepared in PBS for 1 hour at 37°C. Additionally, grids after the above reaction were stained with 1% PTA (phospho-tungstic acid) in water and 5% (w/v) uranyl acetate. Control reactions were performed with omission of the primary antibodies (data not shown).

### 2.7. Ultrastrucutral Localization of U2 snRNA 

At the ultrastructural level, U2 snRNA was detected using a TOR50 probe, chemically labelled at the5′ end and enzymatically labeled at the 3′ end with digoxigenin (F. Hoffmann-LaRoche Ltd., Rotkrenz, Switzerland). Hybridization was performed for 12 hours at 42°C. In order to visualize the probe, sections were incubated with primary mouse antidigoxigenin antibody (F. Hoffmann-Roche) overnight at 4°C. Next, grids were incubated with secondary goat antimouse IgG 15 nm gold (British BioCell) diluted 1 : 50 in 1% BSA prepared in PBS for 1 hour at 37°C. Finally, grids were stained with 1% PTA (phospho-tungstic acid) and 5% (w/v) uranyl acetate. The control reaction was performed using the hybridization with a sense probe.

### 2.8. DNA Detection 

The TdT immunogold technique was performed according to Thiry [34], with modifications. Grids were incubated for 30 minutes at 37°C with the following solution: 20 *μ*M Br-dUTP (Sigma), 200 mM potassium cacodylate, 25 mMTris-HCl, 250 *μ*g/mL bovine serum albumin (BSA), pH 6.6; 5 mM CoCl2, 4 *μ*M dCTP, dGTP, dATP, 2.5 U/*μ*L terminal transferase (Roche). Material was then washed with bidistilled water and incubated successively with PBS, 5% BSA in PBS and again PBS. Incubation with anti-BrdUTP antibody (Roche), diluted 1 : 200 in PBS, was carried out for 1 hour at 37°C. Grids were then washed with PBS and incubated with an antimouse antibodies coupled with 15 nm gold (BioCell). Finally, grids were stained with 5% uranyl acetate.

Ultrastructural analyses were carried out using a Jeol 1010 electron microscope operating at 80 kV.

## 3. Results 

### 3.1. Agyrophylic Nuclear Structures in the Nucleus of Hyacinthus orientalis L.Microspore and Developing Pollen Cells

**Figure 1:**
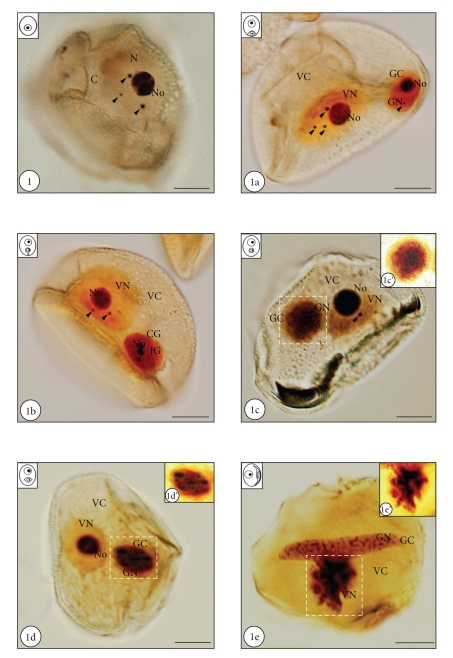
*Hyacinthus orientalis * L. silver nitrate-stained microspore and developing pollen grains (N-nucleus, No - nucleolus, VN-vegetative nucleus, VC-vegetative cytoplasm, GN-generative nucleus, GC-generative cytoplasm). Bars-10 *μ*m. Figure 1. In the nucleus of the microspore there are clearly visible three extranucleolar agyrophylic bodies (arrowheads). Figure 1a. Just after the microspore mitosis, in the vegetative nucleus a nucleolus and three regular bodies can be seen (arrowheads). In the generative nucleus, excluding nucleolus, one regular and strongly agyrophylic body is present (arrowhead). Figure 1b. In both nuclei of young pollen grain nucleolus can be observed. In the vegetative nucleoplasm two silver nitrate-stained bodies are visible (arrowheads). Figure 1c. Pollen grain after detachment of the generative cell from the sporoderm is shown. The vegetative nucleus is with an evident nucleolus and one nuclear body (arrowhead). The generative nucleus exhibits strong Ag-NOR staining, without organized nucleolus and nuclear bodies. Figure 1c’. Magnification of generative nucleus is shown on Figure 1c (area marked with the dashed line). Disperse, highly agyrophylic nucleolar material can be seen in the center of the generative cell. Figure 1d. Premature pollen grain is shown. Only highly agyrophylic nucleolus can be observed in the vegetative nucleoplasm. The generative nucleus shows strong staining. Figure 1d’. Magnification of generative nucleus is shown on Figure 1d (area marked with the dashed line). Strongly Ag-NOR stained nucleolar material is localized in the whole area of the nucleoplasm. Figure 1e. Pollen grain just before antheisis is shown. Highly agyrophylic, flapped-shape vegetative nucleus, without organized nucleolus can be seen. Spindle-shape generative nucleus exhibits the presence of intense silver nitrate staining in the form of irregular clusters. Figure 1e’. Magnification of vegetative nucleus is shown on Figure 1e (area marked with the dashed line). Nucleolar material, in the form of dark clusters, can be seen in the central area of the nucleus

**Figure 2:**
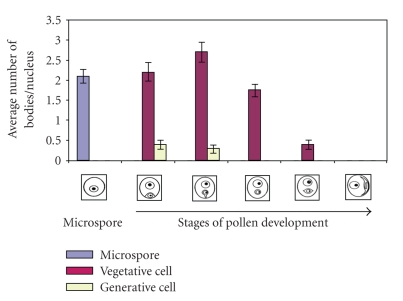
Graph illustrating the average number of silver nitrate-stained extranucleolar bodies in the polarized microspores and differentiating pollen cells. In the microspore the average number of nuclear bodies was 2.2. During the whole course of pollen development the number of these structures was much higher in the vegetative nucleus that in the generative one. Until the separation of the generative cell from the sporoderm, the average number of agyrophylic nuclear bodies in the vegetative nucleus was the highest and reached values of over 2 per nucleus. During later stages of pollen development the average number of nuclear bodies in the vegetative nucleus successively decreased. In the generative nucleus silver nitrate-stained bodies were observed only in young pollen grains and their average number never reached over 0.5 per nucleus. In both nuclei of mature pollen grain no nuclear bodies were indicated. The values represent the average of three replicate experiments; the error bars indicate one standard error of the mean.

Ag-NOR technique revealed the presence of agyrophylic structures in the nucleus of microspore and differentiating pollen cells. In the polarized microspore the places of strong silver impregnation were both the nucleolus and regular nuclear bodies adjacent to the nucleolus or freely localized in the nucleoplasm (Figure 1, arrowheads). The number of bodies in microspores varied from 1 to 4 per nucleus, and its average number was 2.2 per nucleus (Figure 2). The diameter of examined structures was from about 1 to 2 *μ*m. The remaining areas of microspore nucleoplasm exhibited lower affinity for silver nitrate. Just after microspore division, agyrophylic proteins were shown to be present in both pollen cells (Figure 1a). In the vegetative nucleus, strong accumulations of silver ions were observed in the nucleolus as well as in regular shaped nuclear bodies, with a diameter varying from 1 to 2 *μ*m (Figure 1a, arrowheads). At this developmental stage, the number of agyrophylic bodies in the vegetative nucleus varied from 1 to 3 with an average number of over 2 (Figure 2). The remaining areas of the vegetative nucleoplasm showed relatively lower levels of silver impregnation (Figure 1a). In the generative nucleus of young pollen grains, a high affinity for silver nitrate was shown for both, the nucleolus and single, small nuclear bodies. The diameter of these structures varied from 0.8 to 1 *μ*m (Figure 1a, arrowhead) and their average number did not exceed 0.5 per nucleus. It should be noted that, during the whole course of pollen development, the average number of examined structures was significantly higher in the vegetative nucleus than in the generative one (Figure 2). During the progressive separation of the generative cell from the sporoderm (Figure 1b), the strongest silver staining was seen in the nucleolus of both pollen nuclei, and also in nuclear bodies, which were usually observed in the vegetative nucleus (Figure 1b, arrowheads). The diameter of the described bodies was fairly constant during the analyzed period of development and varied from 0.8 to 2.2 *μ*m. The generative nucleoplasm showed a higher concentration of silver ions than the vegetative one, and the segregation of the nucleolus in the generative nucleus was usually observed at this developmental stage (Figure 1b). After the complete detachment of the generative cell from the pollen wall, significant differences in the pattern of silver nitrate staining were observed between both pollen nuclei (Figure 1c), and a successive decrease in the average number of agyrophylic bodies was observed in the vegetative nucleus (Figure 2). Strong affinity for silver ions was shown in the nucleolus, and a few nuclear bodies with a diameter of about 1.5 *μ*m were present in the vegetative nucleus (Figure 1c, arrowhead). In the generative nucleus, with highly condensed chromatin at this period of pollen development, agyrophylic proteins were localized through the whole area of the nucleoplasm. Noticeably, the affinity of the generative nucleus to silver nitrate was comparable with the affinity of the vegetative nucleolus at this developmental stage (Figure 1c). The nucleolar, highly agyrophylic material was scattered through the generative nucleoplasm (Figures 1c and 1c’). Moreover, from this developmental stage, agyrophylic nuclear bodies were no longer observed in the generative nucleus (Figure 2). In the premature pollen grain, strong silver impregnation was observed in the nucleolus of the vegetative nucleus and in the different areas of the generative nucleoplasm (Figures 1d and 1d’). Final stages of pollen development were also accompanied by a complete lack of silver stained nuclear bodies in both pollen nuclei (Figure 2). Just before anthesis, both pollen nuclei showed significant differences in silver staining localization pattern (Figure 1e). In the vegetative nucleus, which exhibits the strongest condensation of chromatin at this developmental stage and a characteristic flapped shape, agyrophylic proteins were mainly localized in the central areas of the nucleoplasm (Figure 1e’). In turn, in the generative nucleus silver staining was observed in the form of irregular clusters present in the whole area of the nucleus (Figure 1e).

### 3.2. Accumulation of Splicing snRNPs in Nuclear Bodies of Microspore and Developing Pollen Cells 

**Figure 3:**
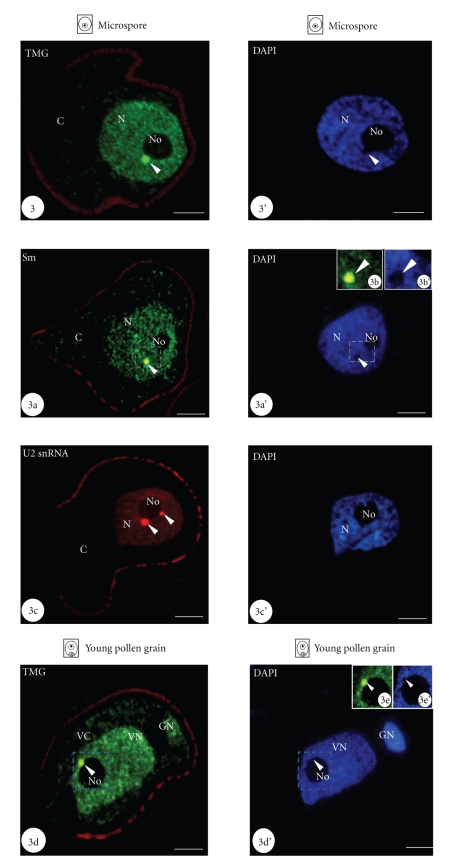

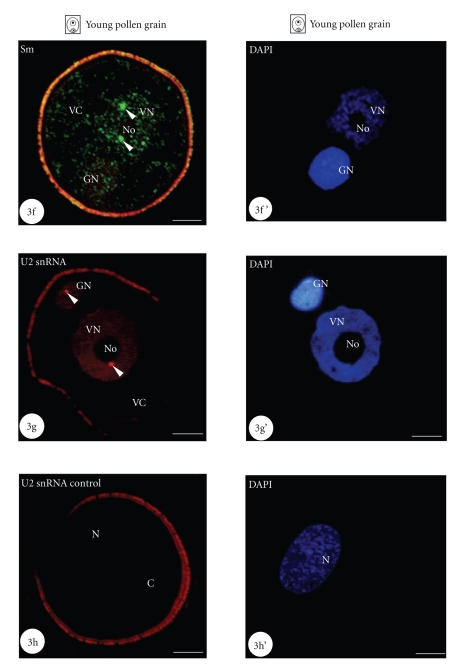
Concentration of snRNP-indicating molecules in nuclear bodies of microspore and young pollen cells showed using immunofluorescence techniques (N-nucleus, No - nucleolus, VN-vegetative nucleus, VC-vegetative cytoplasm, GN-generative nucleus, GC-generative cytoplasm). Figures 3’–3g’. DAPI staining of given microspores and pollen grains are shown. Bars-10 *μ*m. Figure 3. Polarized microspore is shown. Immunofluorescence localization of TMG snRNA indicating its high accumulation in the nuclear body (arrowhead) adjacent to the nucleolus is shown. Figure 3a. Nuclear body containing high levels of Sm proteins (arrowhead) can be seen in the microspore nucleoplasm. Figures 3b and 3b’ show the magnification of the area marked with the dashed line on Figures 3a and 3a’. No DNA labelling can be observed in the area of the nuclear body (arrowheads). Figure 3c. High concentration of U2 snRNA is visible in two nuclear bodies (arrowheads) adjacent to the nucleolus in microspore nucleus. Figure 3d. Pollen grain just after microspore mitosis is shown. Nuclear body containing high levels of TMG snRNA is indicated in the vegetative nucleoplasm (arrowhead). Figures 3e and 3e’ show the magnification of the area marked with the dashed line on Figures 3d and 3d’. The lack of DNA can be observed in the nuclear body area (arrowheads). Figure 3f. Young pollen grain is shown. Two Sm-rich nuclear bodies are present in the vegetative nucleus (arrowheads). Figure 3g. In the vegetative and generative nucleus of young pollen grain one nuclear body, containing U2 snRNA is indicated (arrowheads). Figure 3h. Control reaction of fluorescence in situ hybridization performed with the U2 snRNA sense probe (N-nucleus, C-cytoplasm) is shown. No labeling can be seen in the nucleus of polarized microspore. Figure 3h’. DAPI staining of microspore is shown on Figure 3h.

 Using immunocytochemistry and in situ hybridization we showed a specific concentration of snRNP-representing molecules in nuclear bodies of *Hyacinthus orientalis * L. microspore and differentiating pollen cells. In the microspore, the immunofluorescence of TMG snRNAs was uniformly located in the nucleoplasm as well as in the regular nuclear bodies, often adjacent to the nucleolus (Figure 3, arrowhead). The nucleolus, however, showed no fluorescence. In the microspore cytoplasm only a slight labelling was seen (Figure 3). Sm proteins were also shown to be highly concentrated in nuclear bodies of microspore (Figure 3a). Moreover, the area of the nuclear body was usually devoid of DNA staining (Figures 3a, 3b and 3b’, arrowheads). A lower level of the homogeneous fluorescence, indicating Sm proteins, was observed in the microspore nucleoplasm. In the microspore cytoplasm, a very low signal was present (Figure 3a). Fluorescence in situ hybridization to detect U2 snRNA also showed the specific localization of this molecule in regular nuclear bodies present in the microspore nucleus (Figure 3c, arrowheads). In many cases, these U2 snRNA-rich structures were adjacent to the nucleolus periphery. In the surrounding nucleoplasm, the level of fluorescence was significantly lower. In the nucleolus as well as in the microspore cytoplasm, there was no hybridization signal (Figure 3c).

In the nuclei of young pollen cells, we also showed the presence of nuclear bodies containing high levels of splicing snRNPs (Figures 3d–3g, arrowheads). In both nuclei of newly formed pollen grain, TMG snRNAs were shown to be present in the whole area of the vegetative and generative nucleoplasm. However, the highest concentration was found in nuclear bodies, usually present in the vegetative nucleus and often adjacent to the nucleolus (Figure 3d, arrowhead). The nucleolus was free of labelling. Similar as in the microspore nucleus, in the area of the nuclear body in the vegetative nucleus no DNA was detected (Figures 3d, 3e and 3e’, arrowheads). Immunofluorescence localization of Sm proteins in young pollen cells showed their presence in both pollen nuclei (Figure 3f). The signal was detected both in the pollen nuclei as well as in the cytoplasm of the vegetative and generative cells of young pollen grains. Usually in the vegetative nucleus, 2 or 3 nuclear bodies covered with a strong signal were observed (Figure 3f, arrowheads). Just after microspore mitosis, the hybridization signal indicating the presence of U2 snRNA was also observed in both pollen nuclei. However, the highest levels were found in nuclear bodies (Figure 3g, arrowheads). It should be noted that U2 snRNA-rich nuclear bodies present in the vegetative nucleus were usually larger and much more numerous than in the generative nucleus. Fluorescence in situ hybridization control experiments were performed using U2 snRNA sense probe and showed no labeling of microspore nucleus (Figures 3h and 3h’). Control reactions for immunofluorescence experiments were performed by omitting the primary antibodies and showed no fluorescence signal in examined cells (data not shown).

**Figure 4:**
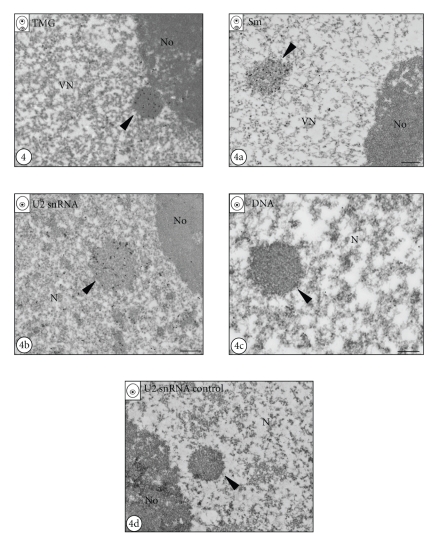
Immunogold localization of snRNP-indicating molecules in nuclear bodies of microspore and vegetative cell of young pollen grain (N-nucleus, No - nucleolus, VN-vegetative nucleus). Bars-0.5 *μ*m. Figure 4. Immunogold labelling of TMG snRNA in the vegetative nucleus of young pollen grain is shown. Strong labelling can be observed in the nuclear body (arrowhead) localized in close proximity to the nucleolus. Figure 4a. Ultrastructural Sm protein localization in the vegetative nucleus of young pollen grain is shown. Numerous gold particles are concentrated over the nuclear body (arrowhead). Figure 4b. In situ hybridization to U2 snRNA is shown. The presence of U2 snRNA over the nuclear body can be seen in the microspore nucleus (arrowhead). Figure 4c. DNA labelling using TdT technique in polarized microspore nucleus (N-nucleus) is shown. No labelling can be seen in the nuclear body (arrowhead). Figure 4d. Control reaction of in situ hybridization performed using U2 snRNA sense probe (N-nucleus, No-nucleolus) is shown. No gold particles can be seen over the nucleoplasm and nuclear body (arrowhead) localized in close proximity to the nucleolus.

Strong accumulations of snRNP-representing molecules using immunofluorescence and FISH techniques were also confirmed at the electron microscope level (Figures 4–4b). Immunogold localization of TMG snRNA in the microspore (data not shown) and the vegetative nucleus of the young pollen grain showed a strong and specific labelling of nuclear bodies (Figure 4, arrowhead). In the surrounding nucleoplasm, the level of the labelling was significantly lower. The nucleolus was devoid of labelling (Figure 4). Gold particles, indicating the presence of Sm proteins were also highly concentrated in the nuclear bodies of microspore (data not shown) and young pollen cells (Figure 4a, arrowhead). Less numerous, dispersed gold particles were also observed over the nucleoplasm of pollen cells. In the nucleolus the lack of colloidal gold was observed (Figure 4a). In situ hybridization of U2 snRNA at EM level showed high levels of this molecule in nuclear bodies of microspore (Figure 4b) and both pollen cells (data not shown). Dispersed gold particles, showing U2 snRNA were also observed across the chromatin of microspore (Figure 4b) and pollen nuclei (data not shown). Additionally, DNA labelling at the ultrastructural level using the TdT technique confirmed the lack of DNA in nuclear bodies of microspore (Figure 4c) and both pollen cells (data not shown). There were no gold particles over the nucleus of polarized microspore after in situ hybridization control experiments, performed using an U2 snRNA sense probe (Figure 4d). Control reactions, performed for all antigens by omitting the primary antibodies, did not show any labelling of neither microspore nor pollen cells (data not shown).

## 4. Discussion

In the present work we have shown the presence of agyrophylic proteins in the nucleus of microspore and developing pollen cells in *H. orientalis *. Silver staining of both differentiating *H.o. * pollen nuclei revealed significant changes in the pattern of silver impregnation in the vegetative and generative nuclei. In the nucleoplasm of both pollen nuclei, a successive increase of silver staining intensity was observed as pollen developed. However the process was observed earlier and was more noticeable in the generative nucleus than in the vegetative nucleus. Just before anthesis in both pollen nuclei, the intensity of silver staining of nucleoplasm was the highest. The most agyrophylic structure in both pollen nuclei of developing was the nucleolus. In the vegetative nucleus a well-organized nucleolus was observed during most of stages of pollen development, except at the end of maturation, when its segregation and disassembly occurred. In the generative nucleus, the nucleolus was observed only until the separation of the generative cell from the sporoderm. During the later stages of pollen development, a segregation and subsequent disintegration of the generative nucleolus was observed. Just before anthesis, most of chromatin from the vegetative nucleus was strongly condensed and agyrophylic proteins were uniformly localized in the flopped-shape nucleus. At this time of pollen development, the generative nucleolus was not observed and agyrophylic proteins were present between areas of condensed chromatin. Changes in silver staining pattern observed during *Hyacinthus orientalis * L. pollen cells differentiation strongly reflect the changes in chromatin condensation state as well as in the organization of the nucleolus during pollen development in this species, described firstly by Bednarska and Górska-Brylass [30]. It seems that observed nuclear changes clearly illustrate the state of gene activity of both pollen cells during their differentiation, which was also described in many other plant species [35–37]. However, the silver impregnation pattern observed in the nuclei of the mature pollen grain, which was shown to be transcriptionally silent [29, 31], was characteristic and may reflect the specific organization of nucleolar material in both pollen nuclei. The lack of the nucleolus in the generative cell just before anthesis was shown to be accompanied by the presence of RNP particles at the periphery of condensed chromatin clumps [30]. This study was carried out using EDTA staining according to Bernhard [38]. In turn, in the vegetative nucleus, similarly to the generative nucleus, RNP particles were localized mainly in the perichromatin regions of the nucleus [30]. Given these data, we assumed that the irregular pattern of silver impregnation is specific and indicates the agyrophylic proteins present in these RNP-positive areas of both pollen nuclei.

 It is known that Cajal bodies (CBs) contain nucleolar proteins of the agyrophylic type; thus the Ag-NOR technique is also one of the methods for their identification in different cell types, including plant cells [39–41]. Indeed, the size and shape of these nuclear structures, as well as the localization in the nucleus, strongly suggest that they are Cajal bodies. Under the electron microscope we observed nuclear bodies as packed, coiled thick threads freely localized in the interchromatin region of the nucleus or adjacent to the nucleolus, which strongly resemble the ultrastructure of Cajal bodies [4, 7, 18, 42]. Additional evidence that the identified structures belong to the Cajal bodies class was the specific and strong accumulation of snRNP-related molecules in their area. At the level of fluorescence and electron microscope we showed that snRNP-specific particles accumulate in round-shaped nuclear bodies, often adjacent to the nucleolus, the diameter as well as the ultrastructure of which strongly corresponds to the Cajal bodies. Given all these data, we assumed that the agyrophylic bodies observed in *Hyacinthus orientalis* L. microspore and developing pollen cells correspond to Cajal bodies and that the Ag-NOR technique can be used as an excellent tool for quantitative analysis of these structures during pollen development.

Analysis of the average number of CBs found in developing microspores and pollen grains in *H. orientalis * indicated its strong positive correlation with the transcriptional activity of these cells, demonstrated in our previous studies [31]. During pollen development, the vegetative cell was shown to be much more active in RNA synthesis than the generative cell. Moreover, the highest levels of transcription in both pollen cells were observed in the young pollen grain, whereas a successive repression of RNA synthesis was found at later stages of pollen development. The mature pollen grain was shown to be transcriptionally silent [29, 31]. Indeed, the obtained results showed that CBs were more abundant in the microspore and the young vegetative cell nuclei. At later stages of pollen maturation, a drastic reduction in the number of CBs was observed in the vegetative cell. In the generative nucleus of *H. orientalis * developing pollen, nuclear bodies were rarely observed (less than 0.5 per nucleus) and only at the young pollen grain stage. We assumed that such a low number of CBs are related to significantly lower levels of RNA synthesis in the generative cell, in comparison with those observed in the microspore or the vegetative cell [31]. It should also be noted that CBs were observed in the generative cell at the stage of young pollen grain only, when the transcriptional activity was the highest [31]. Recently, Segui-Simarro et al. [28] analyzed nuclear bodies in isolated microspores and anther cultures of *Brassica napus * and *Capsicum annuum * L. They showed that in both species, CBs were present in microspores and the vegetative cell of the young pollen grains, just after the first pollen mitosis, but at low amounts. In the mature pollen, NBs were rarely observed. Based on the transcriptional activity of these cells, these results suggest a correlation between the transcriptional activity and the presence of CBs. Additionally, after microspore embryogenesis, which is accompanied by an increase in transcriptional activity, the authors indicated that young proembryos are highly engaged in proliferation and that the diameter as well as the number of CBs per cell increased [28]. In many different types of mammalian cells, CBs were also shown to be related to transcriptional activation, cell cycle progression and metabolic activity [2, 7, 8, 43, 44]. Moreover, nuclear bodies have been suggested as potentially sensitive to changes in proliferative activity also in plant cells [45]. Thus, our results seem to confirm the common opinion that CBs are characteristics for high metabolically active cells and that their number and/or size reflects the transcriptional state of the cell.

 We also revealed a strong positive correlation between the average number of CBs and the levels of splicing machinery elements in the microspore and the differentiating pollen cells, presented in our previous reports [32]. Our studies revealed that the highest levels of TMG snRNA, Sm proteins as well as SC35 splicing factors occur in the microspore and the young vegetative pollen cells, whereas at later stages of pollen development the level of all examined antigens successively decreased and reached its minimum just before anthesis. During pollen grain maturation, the level of splicing machinery elements was much higher in the vegetative cell than in the generative cell [32]. At the microspore stage and in the young vegetative cells, CBs were most abundant. During later stages of pollen development, both the level of splicing machinery elements and the number of CBs successively decreased.

Here, we showed a strong accumulation of TMG snRNA, Sm proteins and U2 snRNA in Cajal bodies using immunofluorescence and immunogold techniques. All examined molecules were also shown to be specific markers of CBs in both the microspore and the differentiating pollen cells. In metabolically active cells an intensification of the RNA synthesis and processing is usually observed and leads to higher levels of transcriptional/splicing machinery elements in these cells. The model of snRNP biogenesis, proposed by Matera [12], assumes that CBs play an essential role in both snRNAs transcription and the final steps of snRNPs maturation. It has been shown that several snRNA encoding genes, including U1 and U2 snRNAs, are spatially adjacent to the CBs periphery [46, 47]. The authors speculated that CBs may be involved in the regulation of snRNAs transcription. More recent results of Darzacq et al. [14] indicated the reason why some snRNPs partially accumulate in Cajal bodies after their return to the nucleus. They identified a novel class of small RNAs, scaRNAs (specific Cajal bodies RNAs), which is specific only for these nuclear structures and function in pseudourydilation and methylation of snRNAs, which are the final steps of snRNP biogenesis [10, 13]. Mature snRNP particles are ready for catalyzing the splicing reaction. Because of the fact that CBs were most abundant at developmental stages during which the levels of snRNPs were the highest, especially in the cytoplasm of both pollen cells [32], we cannot exclude that their presence reflects an intensification of snRNP biogenesis in the microspore and the young pollen cells. Moreover, transcriptional activity of the cell and intensification of snRNP biogenesis are tightly functionally coupled; thus it seems that the model proposed by Matera is especially true and clearly visible in microspores and pollen cells of *H. orientalis*.

The universal nature of these extraordinary nuclear structures was confirmed also by their identification in different types of cells in such evolutionary distant species such as dinoflagellates [48], *Drosophila * [49], *Xenopus * [50], and mammals [9]. CBs have also been observed in microspores and pollen cells in different plant species, for example, in *Brassica napus * [27], olive [21], Douglas fir [25], and larch [24, 26]. Given all these facts, it seems that Cajal bodies are evolutionarily conserved nuclear structures, and in all cases they function as an important element of nuclear gene expression machinery in eukaryotic cells.
